# Annotations

**Published:** 1910-06-04

**Authors:** 


### ANNOTATIONS.

### The Death of Professor Koch.

To his many friends the announcement of Professor Koch's death hardly comes as a great surprise, since it has been well known to them that for some time past the famous bacteriologist's health was precarious in the extreme. He himself was fully conscious of the gravity of his case, and though the end came sooner than was expected, he was assuredly not unprepared for it. His death removes the greatest of German scientists from the serried ranks of German medical men. He was not, it is true, a great physician, although he fulfilled the first requisite towards the attainment of that high position which the Salernian canon laid down, in so far that he was a skilled dialectician and fluent speaker, but he was a great scientist in the fullest sense of the term. Starting professional life as a village doctor, his early struggle was curiously similar to that of the illustrious Jenner—for whom, it is interesting to note, Robert Koch always expressed the most intense admiration—and his startlingly successful career is another proof that the general practitioner, with energy and ability, carries in his pocket the professional equivalent of a field-marshal's bâton. The details of Professor Koch's remarkable life are well enough known to need no explanatory note here, and those who are acquainted with them will not have to be reminded that the keynote of his success was "thoroughness." It is this characteristic of his that may well give us pause in rejecting fully his conclusions with regard to bovine tuberculosis in favour of those now generally accepted by the profession. We are the last in the world to advocate blind respect for authority and the equally unreasoning acceptation of everything that a famous scientist utters, but we confess to a sneaking suspicion that on this question Koch may be right after all, and we believe that we are not singular in maintaining this heresy. But over his bier controversial questions, with which during his eventful life he was so intimately associated, will have a rest. We can only deplore the death of a great master, and those of us who were privileged to know him personally, who loved the quiet thoughtfulness that endeared him to his pupils, the self-sacrificing devotion to duty and to science that was at the same time the admiration and the exasperation of his assistants, and the innate friendliness and lovableness of the man himself, mourn the passing of a good man and friend whose place it will be difficult to fill.

### Medical Men and Slang.

"Alike in Senate and in individual intercourse, let your language be dignified, but not elaborate," said Marcus Aurelius, but members of the medical profession are prone to ignore this wise advice. As young men they talk about "yanking out the lump," or "clearing out the kid's guts"; and then, as old age comes on them, they go to the other extreme and tell the patient that "he is suffering from a febricular affection associated with a hyperæmic condition of the nasal mucosa." Each phase is bad. The first the layman puts down to callousness, the second only brings ridicule to the doctor, generally with an accusation of ignorance. And yet the one is probably the outcome of the other. A man who has allowed himself to drift into a loose way of speaking finds that it is distinctly unpopular with his patients, and in trying to stop it passes insensibly into a Johnsonian phraseology which become more pronounced with age. The lesson of Marcus Aurelius should be learnt in youth, and a studious exclusion of all slang terms from general talk, even when they sound "smart," is the only way to prevent such terms from creeping into the professional conversation between doctor and patient. Slang terms, however pithy, are always out of place, and frequently jarring to the listener and discreditable to the speaker. The older a man gets before he tries to rid himself of the habit the less likely is he to free himself from the accusation of either indifference and ignorance or pomposity, vagueness, and affectation.

### "Le chirurgien malgré lui."

Commandant Michaux, of the Belgian Army, in a volume entitled "Carnet de Campagne au Congo; épisodes et impressions de 1889 à 1897," describes some of the shifts to which officers are at times put in foreign countries and distant colonies where the services of a medical officer cannot be had. He relates how on one occasion he, as a layman, was called upon in an emergency to act as surgeon. "While fighting the Baknas Tombolos one of my soldiers had a finger completely shattered by a ball. To leave him as he was meant gangrene and certain death; but I was at a loss to know what to do to remove his finger. I resolved, therefore, to proceed as follows: Having caused three dead Backnas to be brought into a hut, I removed, with Saudrart's help, from each corpse the finger corresponding to the one of my Haoussa which had been shattered. Then, when I was certain that the operation would proceed with sufficient smoothness, I operated. I was lucky enough to succeed, and in a fortnight the man was completely cured. This good result encouraged me greatly, and after that I continued to act in a similar fashion. As I was not particularly well posted in anatomy, I always commenced, when I had an operation to perform, by holding what I may call a dress-rehearsal on the dead body. But it will doubtless be said that at such a game I must have killed many? No, i' faith: not too many. . . . No doubt also some will ask how it was that I always had chloroform at hand? The answer is simple: I did not send them to sleep. 'Then,' they will say, 'you operated on them while fully conscious?' Exactly so. Was it better to let them die? True they cried out a little at times; but I finished by not hearing them. I even had an advantage over my doctor *confrères* in that so long as my patients cried out I was at least certain that they were not dead!"

